# A case study of the application of AI to early stage drug discovery

**DOI:** 10.1038/s41598-025-32805-1

**Published:** 2025-12-26

**Authors:** Abbi Abdel-Rehim, Larisa N. Soldatova, Ross D. King

**Affiliations:** 1 Department of Chemical Engineering and Biotechnology, University of Cambridge, Cambridge, UK; 2 Department of Computing, University of London, London, UK; 3 Department of Biology and Biological Engineering, Chalmers University of Technology, Gothenburg, Sweden; 4 Department of Computer Science and Engineering, Chalmers University of Technology, Gothenburg, Sweden; 5 The Alan Turing Institute, London, UK

**Keywords:** Chemistry, Computational biology and bioinformatics, Drug discovery

## Abstract

Artificial intelligence (AI) has emerged as a powerful tool in drug discovery, offering the potential to expedite the design of novel therapeutics. This study evaluates the effectiveness of a general-purpose conversational AI, ChatGPT (GPT-4o), in performing three distinct drug discovery tasks, assessing its ability to assist in early-stage molecular ideation and design. In the first task, ChatGPT generated molecules starting from five low-affinity EGFR inhibitors (IC₅₀ values of 10–3.16 µM), which were iteratively optimized in a QSAR model to produce compounds with predicted IC₅₀ values of ~ 10–50 nM. In the second task, de novo design of EGFR inhibitors produced a molecule with a predicted IC₅₀ of 94 nM in a single attempt. In the third task, ChatGPT generated non-covalent MCL1 inhibitors, with a top candidate achieving a docking score corresponding to a 39 nM dissociation constant. Because AI-generated molecules often face synthetic feasibility challenges, we also identified readily available analogues from a chemical vendor. These analogues were evaluated using molecular docking (AutoDock Vina) and QSAR models, with several achieving a promising activity range of 10–100 nM across the three tasks. These results demonstrate that general-purpose AI models like ChatGPT can accelerate early-stage drug discovery by assisting in molecular ideation and candidate prioritization.

**Supplementary Information:**

The online version contains supplementary material available at 10.1038/s41598-025-32805-1.

## Introduction

Artificial intelligence (AI) has emerged as a powerful tool in drug discovery, with the potential to accelerate the design of therapeutic agents. Traditional processes relying on screening and synthesis are resource-intensive and time-consuming. Recent advances in machine learning, particularly large language models (LLMs), have the potential to streamline early-stage drug discovery by automating molecular design^1–3^.

Most generative AI studies rely on specialized models trained on domain-specific data for tasks such as QSAR modelling, molecular docking, or drug repurposing^4–6^. While models like BioGPT and DrugChat excel within their focused domains, they are often limited to specific tasks and require retraining or integration with other tools for broader applications^7–9^. In contrast, ChatGPT is a general-purpose large language model that is immediately accessible and does not require fine-tuning to begin supporting drug discovery tasks. This versatility makes ChatGPT a flexible and cost-effective solution for researchers without deep AI expertise, particularly in early-stage discovery where rapid ideation and broad exploration are crucial. Instead of relying on multiple specialized models, ChatGPT can generate candidate ideas across several objectives, from hypothesis generation and molecular design to strategic planning, serving as a flexible starting point for researchers^10^.

**Fig. 1:**
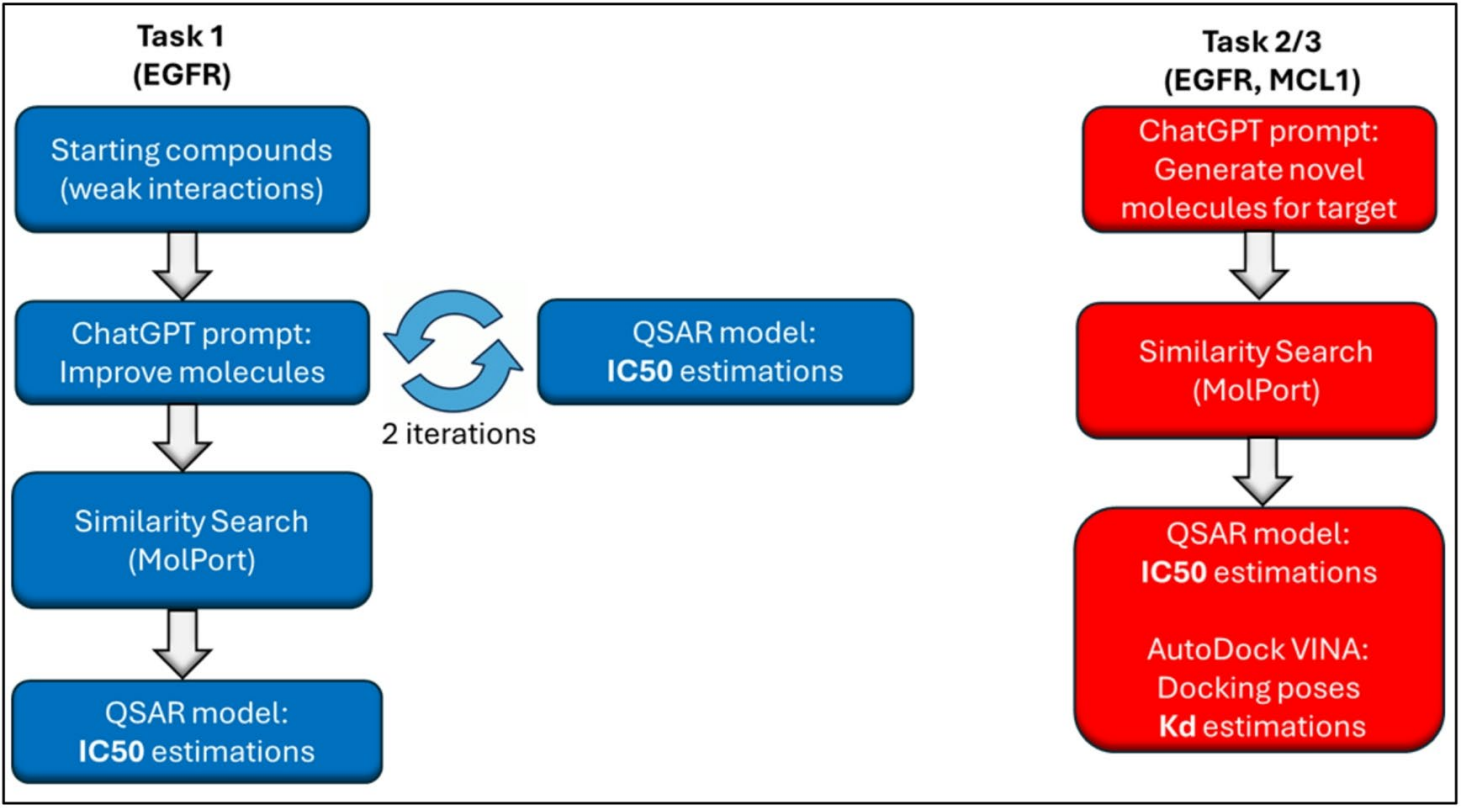
Flowchart describing the two different approaches used in this study. Task 1) Investigate the ability of ChatGPT to generate improved molecules based on weakly binding molecules without mentioning the target (EGFR). Task 2/3) Investigate ChatGPTs ability to generate novel molecules ab initio against two disclosed targets (EGFR, MCL1).

In this study, we investigate the performance of ChatGPT to support early-stage drug discovery across three in silico tasks: QSAR guided design of molecules targeting EGFR, de novo inhibitor design for EGFR, and non-covalent inhibitor design for MCL1 (Fig. 1). Despite existing therapies, both proteins remain active areas of research due to issues like resistance mutations in EGFR (e.g., T790M, C797S) and toxicity or resistance mechanisms in MCL1 inhibition^11–15^. Their extensive structural and functional characterization provides a rich foundation for computational design, making them ideal test cases for evaluating LLM-driven drug discovery.

The goal of this work is not to replace existing workflows, but to assess how a versatile “off the shelf” AI can spark creative hypotheses and propose viable drug candidates before substantial resources are committed. To ensure practical feasibility, we paired ChatGPT’s molecule suggestions with similarity searches in MolPort to identify commercially available analogues suitable for expedient experimental testing.

The first task involved using ChatGPT to design molecules for EGFR from a starting point of five low-affinity compounds (IC_50_ values of 10–3.16 µM), without providing any information about the target protein. After two QSAR-guided iterations, the generated molecules had estimated IC_50_ values of ~ 10–50 nM, with corresponding analogues in the range of 20–50 nM, demonstrating successful optimization even without explicit structural context. In the second task, ChatGPT was informed that the target was EGFR and tasked with generating novel EGFR inhibitors in a single attempt, leveraging training-derived associations from its large corpus to propose relevant compounds. Some generated molecules had predicted IC₅₀ values around 100 nM, and the top analogue achieved a predicted IC_50_ value of 56 nM with an estimated dissociation constant of 10 nM. In the third task, focused on MCL1, the top generated molecule achieved a QSAR predicted affinity of 39 nM, though its analogues were not performing as well. The top performing analogue, featuring an added linker, achieved a docking score corresponding to an activity of 108 nM (k_d_) and a QSAR predicted activity of 46 nM (k_i_).

These findings demonstrate that output from ChatGPT can support early-stage drug discovery workflows. While not specifically trained for molecular design, ChatGPT generated candidate structures that were used to explore ideation and preliminary optimisation of compounds. As expected, the molecules generated by ChatGPT may not be directly suitable for experimental testing and generally require further in silico refinement or analogue-based exploration, as demonstrated in this study. Nonetheless, this work highlights the potential of accessible, general-purpose LLMs to complement traditional approaches and specialized models, particularly when rapid hypothesis generation and broad molecular exploration are needed.

## Materials and methods

### Compound generation and dataset collection

For this study, ChatGPT-4o was used to generate novel molecular candidates for EGFR and MCL1, with the lighter GPT-4o mini model employed in Task 1 to encourage greater creativity. The generated compounds were represented as SMILES strings, and all valid entries were processed for further analysis using RDKit^16^. EGFR (IC_50_) and MCL1 (K_i_) data was downloaded from ChEMBL. Small molecules were retained for training the QSAR models: <1.1 kDa for EGFR, and the less stringent < 2.5 kDa for the smaller MCL1 dataset.

### QSAR model development and evaluation

IC₅₀ and K_i_ values for EGFR and MCL1, respectively, were extracted from the ChEMBL database (version 35), and pChEMBL values were calculated as –log₁₀(activity) for use in QSAR modeling. Molecular structures were represented as MACCS fingerprints generated from SMILES codes using RDKit (MACCSkeys). For EGFR, a Random Forest model (scikit-learn implementation) with 100 trees and default parameters was trained on the dataset, while the smaller MCL1 dataset (~ 1,000 measurements) was modeled using a Gradient Boosting Regressor with 100 trees and a maximum depth of 10^17^. Model performance was evaluated using 10-fold cross-validation. For EGFR, the results were Spearman *R* = 0.76 ± 0.03, MSE = 0.76 ± 0.09, and RMSE = 0.87 ± 0.05. For MCL1, the corresponding values were Spearman *R* = 0.84 ± 0.04, MSE = 0.38 ± 0.04, and RMSE = 0.61 ± 0.03.

### Molecular docking

Receptor Preparation: The geometric center of the bound ligand was measured to determine the coordinates for the center of the binding site. The receptor structures (proteins) were obtained from the RCSB Protein Data Bank (PDB code 6FS1 for MCL1 and 3BEL for EGFR). Water molecules were removed, and the protein was saved in PDB format. It was then processed using the prepare_receptor4.py script from AutoDock Tools to generate a PDBQT file ready for docking.

Ligand Preparation: For all tasks, ligands were generated using RDKit. The ligands were processed as follows: hydrogens were added, the molecule was embedded, and MMFF optimization was performed. The final molecules were written to PDB format and converted to PDBQT format using prepare_ligand4.py from AutoDock Tools for docking.

Docking Procedure: Docking simulations were performed using AutoDock Vina^18,19^. The search space was defined as a cube with dimensions 20 × 20 × 20 Ångströms, centered around the center coordinates of the binding site. The docking parameters were set to ‘--num_modes “3"’, ‘--exhaustiveness “20"’ and ‘--energy_range “7"’ to balance search thoroughness and speed. The resulting binding affinities were reported as estimated K_d_ values and used to assess the compounds. Image generation of molecular structures was done in ChimeraX^20^.

### Similarity search

A Chemical Similarity Index was used to perform similarity searches on the MolPort platform^21^. The top 10 or 5 most similar compounds to the selected AI-generated molecules were retrieved, and their predicted binding affinities were evaluated using the same QSAR model.

## Results

### Task 1: drug design for EGFR using iterative procedure

For task 1, three initial batches of compounds were provided to ChatGPT, each containing five structurally diverse molecules with moderate activity values (pChEMBL: 5.01–5.47, corresponding to IC_50_ values of ~ 3–10 µM). These batches included scaffolds and functional groups common in kinase inhibitors, such as halogenated aromatics, heterocycles, and polar groups like nitriles and hydroxyls. The molecular structures and SMILES codes for these compounds are detailed in Table S1 and S2. This diversity provided a broader chemical space for ChatGPT to explore.

Three independent experiments were conducted to evaluate ChatGPT’s ability to iteratively optimize molecular designs for EGFR using QSAR-guided predictions. The QSAR model used for evaluation was built using available small-molecule IC_50_ measurements against EGFR in ChEMBL (see methods). Each experiment consisted of two iterations, and the top-predicted molecules after the final iteration (iteration 2) for each batch and experiment are summarized in Table 1 and Table S3 and S4.

**Table 1:** Predicted activities (IC_50_ values with corresponding pChEMBL values in brackets), for the top generated molecule from each randomly selected batch of five start molecules, across three independent experiments.

	Exp. 1	Exp. 2	Exp. 3
Batch 1	32 nM (7.492)	330 nM (6.481)	52 nM (7.281)
Batch 2	52 nM (7.287)	48 nM (7.315)	24 nM (7.624)
Batch 3	105 nM (6.979)	106 nM (6.947)	11 nM (7.950)

The molecular structures of the top predicted compounds from each batch and experiment (9 compounds in total) are shown in Table S3, with corresponding SMILES strings listed in Table S4. These molecules show substantial improvements in predicted binding affinities over the starting compounds.

Notably, the generated molecules showed structural diversity across scaffolds and functional groups, including halogenated aromatics, heterocycles, and polar substituents, contributing to improved binding affinity predictions.

ChatGPT appears to extract key scaffolds from the poorly performing initial molecules. Several generated molecules share features with known EGFR inhibitors, including 4-quinazolineamine and benzimidazole derivatives, as well as halogenated aromatic rings, all of which align with known EGFR inhibitor pharmacophores and indicate that ChatGPT’s generative process reflects established structure-activity relationships^22–25^.

Some molecules feature unconventional elements for EGFR inhibitors, such as a thiourea-containing heterocycle (mol 1. 6), a thiophene with multiple substitutions including bromine and a fluoro group (mol 1.7), and a sulfone group (mol 1.9). These atypical structures increase chemical diversity and may enable exploration of novel binding modes or resistant EGFR variants.

This combination of structurally familiar and unusual scaffolds highlights ChatGPT’s ability to leverage known features while proposing novel approaches to molecular design.

### Similarity search and validation of predicted molecules

A similarity search of the MolPort catalog was performed to assess the practical utility of the GPT-generated molecules (see methods). This search identified commercially available compounds most similar to the highest-affinity predictions from the final QSAR-guided iteration. Top-ranked analogues were evaluated with the QSAR models to confirm strong predicted affinities. Three identified molecules belonged to the class of 4-quinazolineamine derivatives, a well-established scaffold for EGFR inhibition.

All three compounds are experimentally confirmed EGFR binders, supporting the GPT-designed molecules as valid starting points for optimization. The structural similarity between the identified compounds and GPT predictions shows that a general-purpose model can converge on known pharmacologically relevant scaffolds without target-specific training. The structures of these identified 4-quinazolineamine derivatives, along with their respective predicted binding affinities, are shown in Table 2.

**Table 2:**
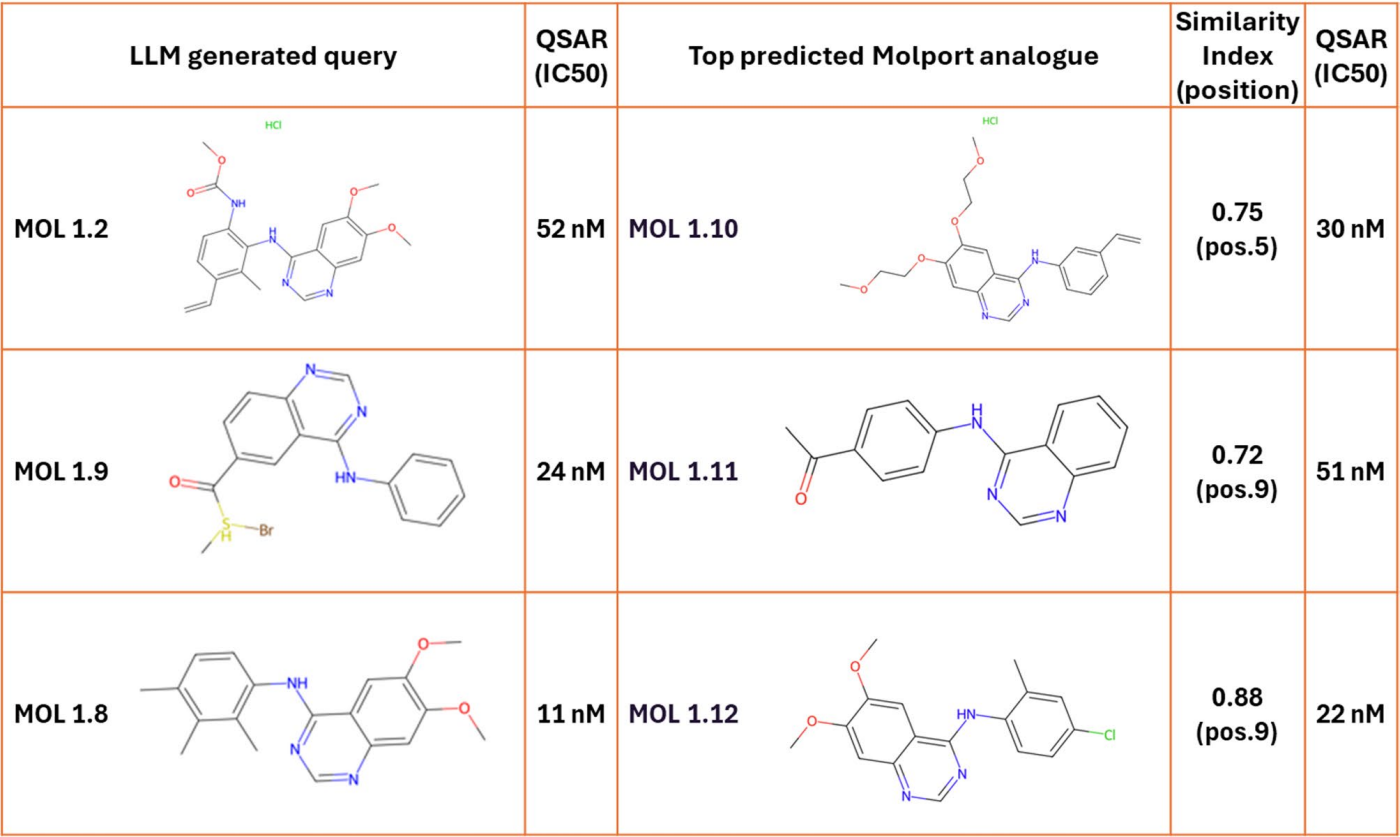
Molecules identified as 4-quinazolineamine-based derivatives derived from similarity searches in molport based on queries of top GPT-designed molecules.

All three analogues (MOL 1.10, 1.11 and 1.12) have previously been confirmed to bind EGFR.

### Non-quinazoline derivatives identified via similarity searches

Beyond quinazolineamine derivatives, the MolPort search revealed structurally diverse non-quinazoline compounds resembling the GPT-designed molecules. Four compounds (MOL 1.16–1.19, Table 3) represent opportunities for scaffold hopping in kinase inhibitor development.

Molecules 1.16–1.18 lack prior reports of EGFR inhibition, making them interesting candidates for experimental validation. Molecule 1.19 is linked to a patent for Protein Kinase C (PKC) inhibitors^26^. Since PKC shares an ATP-binding domain with EGFR, cross-reactivity is possible, but kinase-specific differences underscore the need for careful evaluation.

Molecules 1.16–1.18 feature unique structural motifs. Molecule 1.17 originates from a malaria drug discovery library (CHEMBL5016744). Its structural motifs may offer interesting opportunities for kinase inhibition.

**Table 3:**
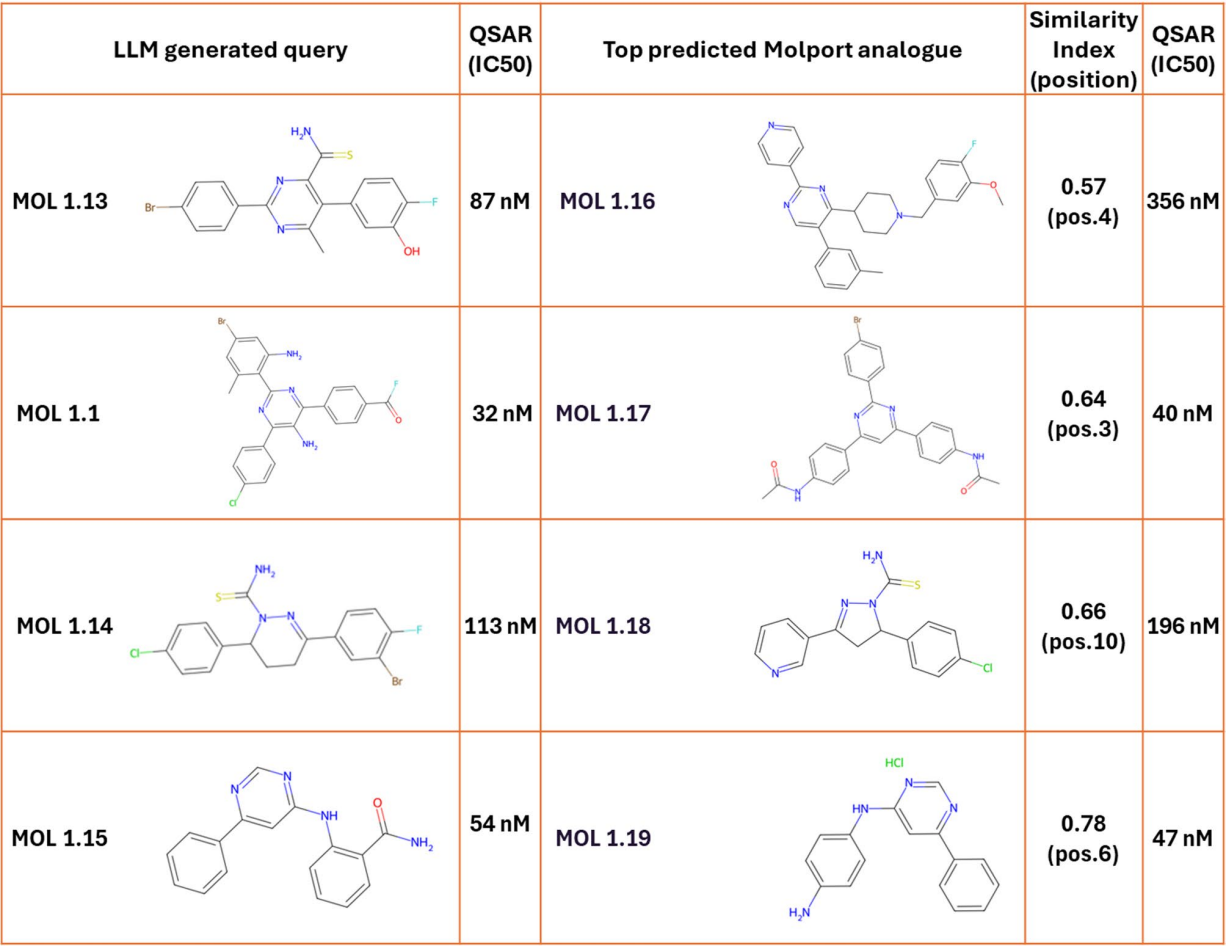
Structures of non-quinazoline-based molecules MOL 1.16–1.19, along with molecules identified through a molport similarity search, the chemical similarity index for the analogues and their rankings amongst the top 10 similar molecules in molport (position), and QSAR predicted activities.

SMILES codes for the molecules can be found in Table S4.

In conclusion, ChatGPT generated molecules that improved affinity toward EGFR. Similarity searches demonstrated that these outputs could guide the selection of viable candidates for experimental follow-up, addressing limitations in synthesisability. The system proposed both known scaffolds, such as 4-quinazolineamine derivatives, and novel hinge-binding structures suitable for ATP-competitive kinase inhibition. Compounds such as 1.15–1.17 highlight opportunities for testing, while 1.19 may exhibit cross-kinase activity. Overall, these results illustrate ChatGPT’s ability to expand chemical space exploring both familiar and novel scaffolds, supporting early-stage drug discovery.

### Task 2: design of novel EGFR inhibitors and identification of potent hits via similarity search

In task 2, ChatGPT, prompted as a drug development expert, designed five novel molecules with strong predicted affinity for EGFR. The aim was to propose structurally distinct starting points emphasizing novelty, drug-likeness, and synthetic feasibility (Appendix A).

For each of the three experiments, the top QSAR predicted molecule had estimated IC_50_ values of 94 nM, 116 nM, and 338 nM (Table 4). Their SMILES were used in MolPort to identify the 10 most similar commercially available analogues per molecule, whose predicted binding affinities were also evaluated (Appendix B). The top analogues showed predicted IC_50_ values of 55 nM, 56 nM, and 165 nM for each of the three experiments (Table 4), demonstrating that similarity searches can uncover structural diverse compounds with comparable or even improved predicted binding affinities.

Top analogues were then docked using AutoDock Vina to assess EGFR interactions. Docking estimated binding affinities (K_d_) of 1.35 µM, 10 nM, and 77 nM for the leading compounds from each experiment, indicating strong binding affinity (Table 4).

**Table 4:**
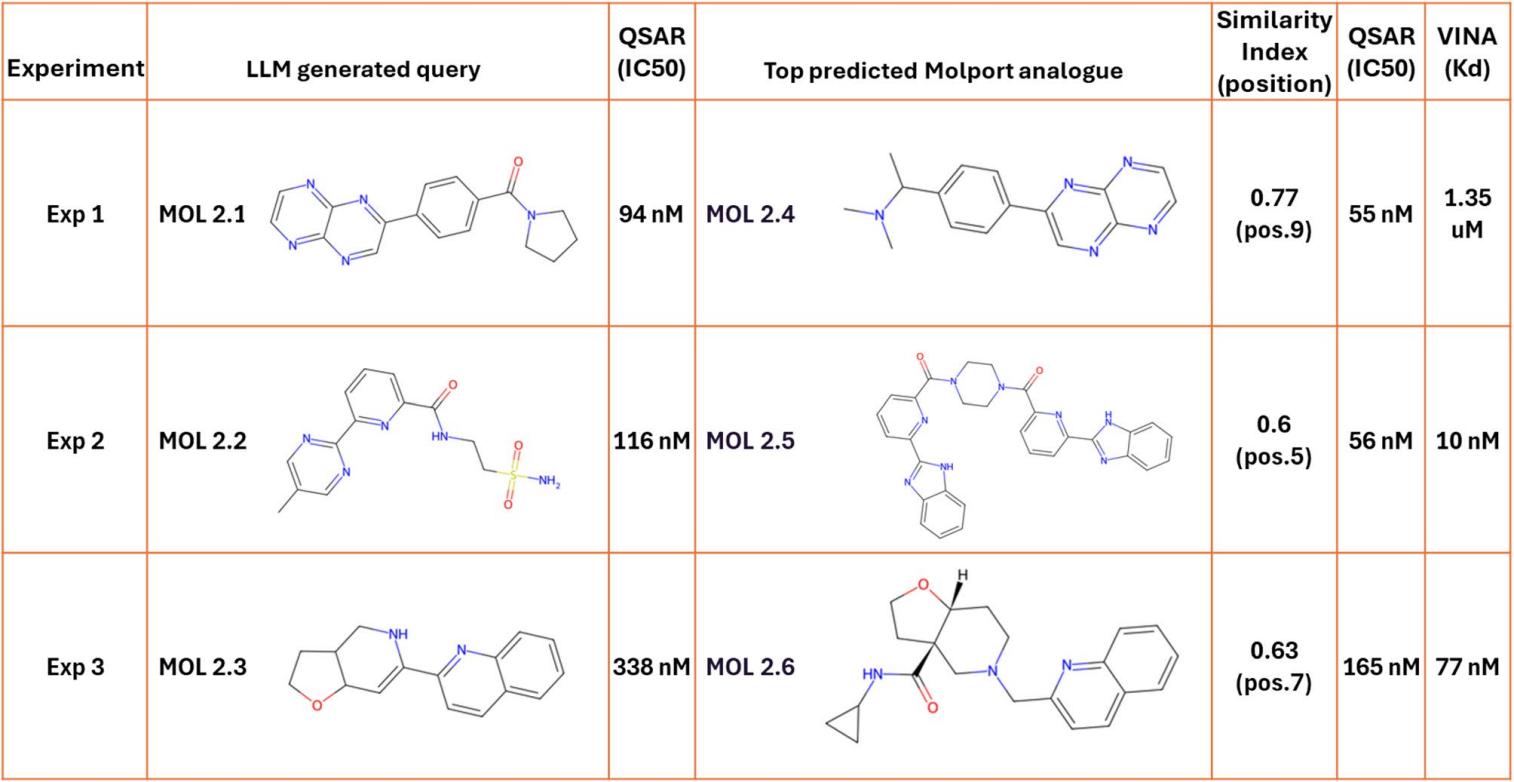
Summary of the top ChatGPT-generated EGFR inhibitor from each of three experiments in task 2, and their QSAR-predicted IC_50_ values.

The table also includes the highest-scoring Molport analogues for each compound, their respective chemical similarity index, their rankings amongst the top 10 similar molecules in MolPort (position), along with their QSAR-predicted IC50 and docking-estimated Kd values. SMILES codes for molecules can be found in Table S5.

**Fig. 2:**
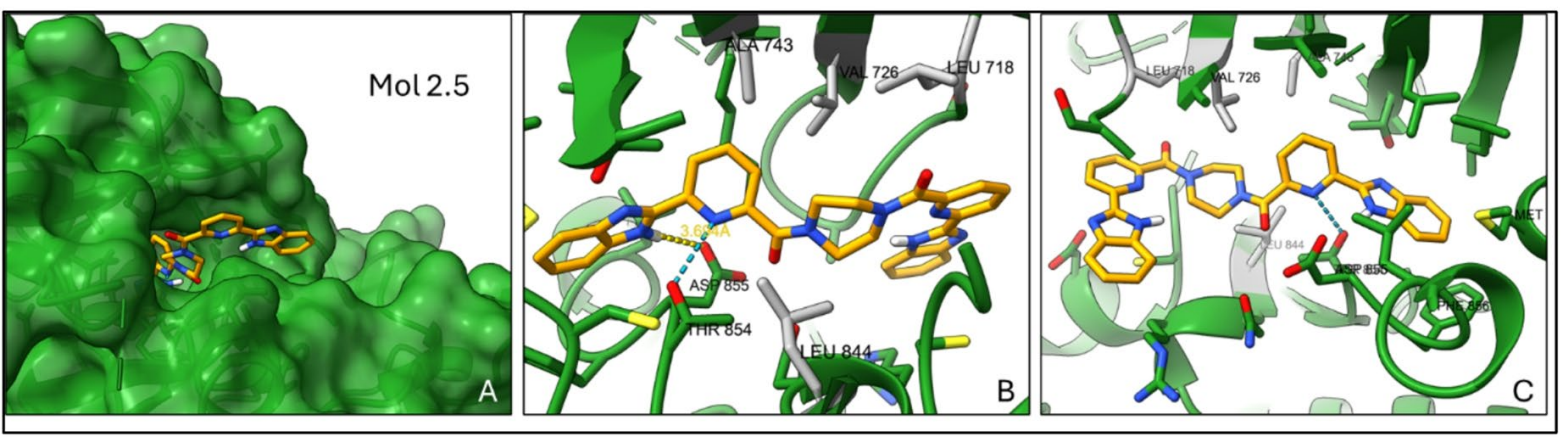
Docking simulation of molecule 2.5 in the EGFR binding pocket (3BEL). (**A**) Surface representation of the EGFR structure with molecule 2.5 (omilancor) entering the binding pocket. (**B**) Close-up view of the hydrophobic residues (ALA743, VAL726, LEU718) just past the entrance. Also highlighted are the hydrogen bond with Thr854 (blue dashes) and the estimated distance of 3.694 Å for a potential hydrogen bond with Asp855 (yellow dashes). (**C**) Full view of the molecule extending deep into the EGFR binding pocket, showcasing its unique conformation and interactions with key residues, including hydrophobic and hydrogen bond interactions.

Remarkably, the best-performing molecule, Mol 2.5, corresponds to omilancor, an FDA-approved compound with no reported EGFR activity^27^, achieving an estimated K_d_ of 10 nM. Resultant docking poses suggest that its extended conformation reaches deep into the EGFR binding pocket (Fig. 2). The benzimidazole group at one end of the molecule is positioned close to the hydrophobic residues Phe856 and Met766, important contacts in known EGFR inhibitors^28^, while the benzimidazole group on the opposite end bends onto the pocket surface, potentially stabilizing the ligand through hydrophobic interactions. The pyridine may form a potential hydrogen bond with Thr854, a key residue involved in ligand binding. The secondary amine lies close to Asp855 (3.69 Å) and Thr845 (3.28 Å), suggesting additional hydrogen bonding^29^. The central ketone-piperazine-ketone motif bridges hydrophobic and polar regions, providing amphiphilic flexibility. Piperazine has been employed in several EGFR inhibitors to improve solubility and selectivity^30,31^. It is important to note that our docking experiments do not account for explicit water molecules, protein flexibility and solvent effects which could influence binding predictions. Overall Mol 2.5’s extended, mirror-like arrangement of benzimidazoles appear to maximize interactions within the pocket, suggesting a favourable fit and a promising candidate for further optimization.

Overall, ChatGPT-generated molecules in task 2, combined with similarity searches, produced structurally diverse, readily accessible candidates with competitive predicted affinities for EGFR inhibition. For an overview of all generated compounds and their predicted activities, as well as the top 10 similar molecules retrieved from MolPort with their similarity scores, see Appendices A and B.

### Task 3: de novo design of non-covalent inhibitors for MCL1

In task 3, ChatGPT was used to design non-covalent inhibitors for MCL1, a BCL2 family protein linked with poor tumor outcomes^32^. Five experiments were conducted, each proposing five molecules. From these experiments, six compounds achieved estimated binding affinities of -9 kcal/mol (K_d_=250 nM) or better (Table S6).

Similarity searches against MolPort retrieved the top five most similar compounds for each of the six molecules (Appendix B). Top ranked analogues showed estimated binding affinities of -10.5 to -9.0 kcal/mol (K_d_~10 to 250 nM) (Table 5).

**Table 5:**
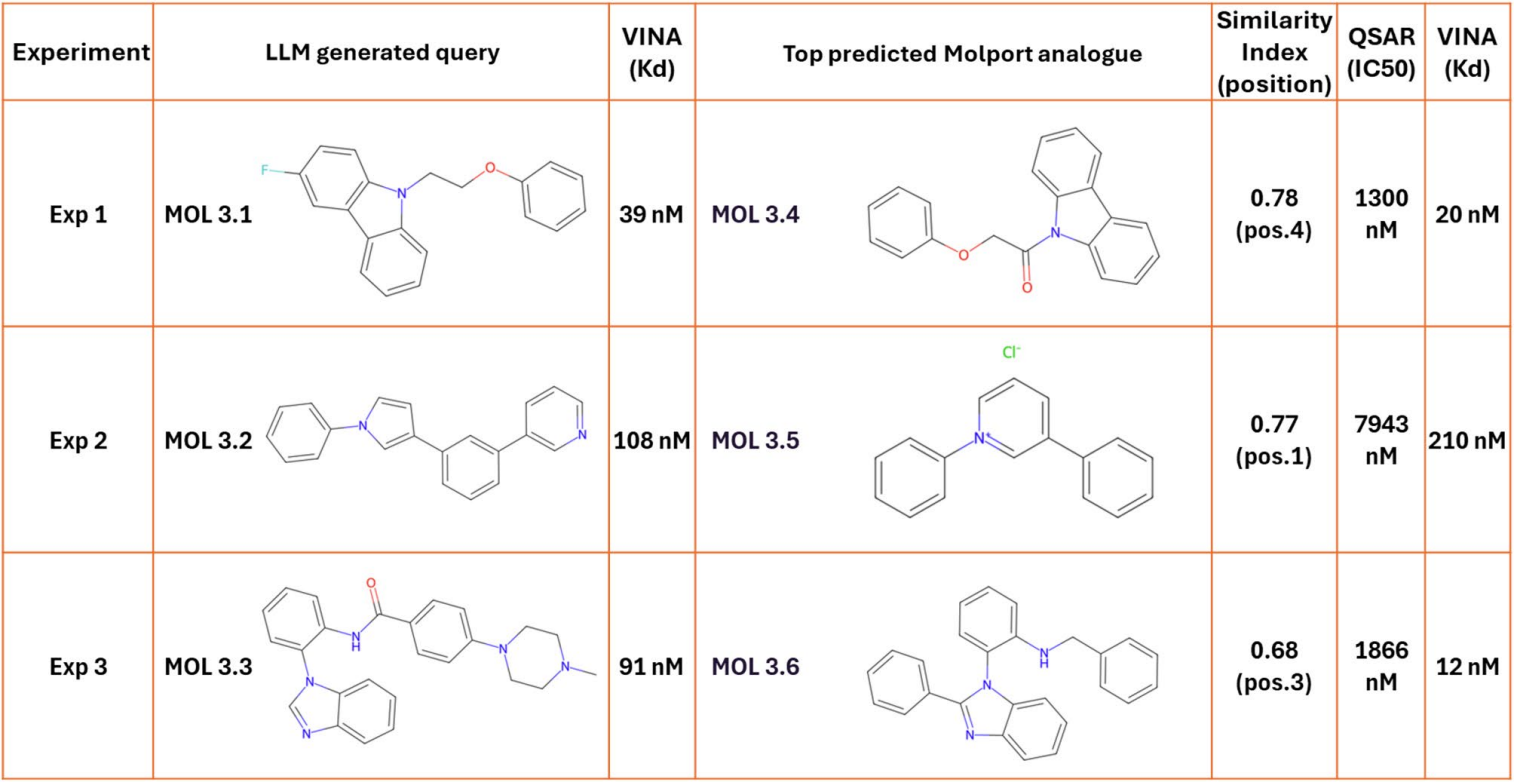
Potential candidates for MCL1 binding.

The table presents the top three generated molecules (mol 3.1, 3.2, and 3.3) from three independent experiments along with their estimated affinities (K_d_) from VINA. The corresponding top hits from the MolPort database (mol 3.4, 3.5, and 3.6) are shown alongside their chemical similarity index, ranking amongst the top 5 similar molecules in MolPort (position), QSAR-predicted activities (K_i_), and VINA affinity estimations (K_d_).

**Fig. 3:**
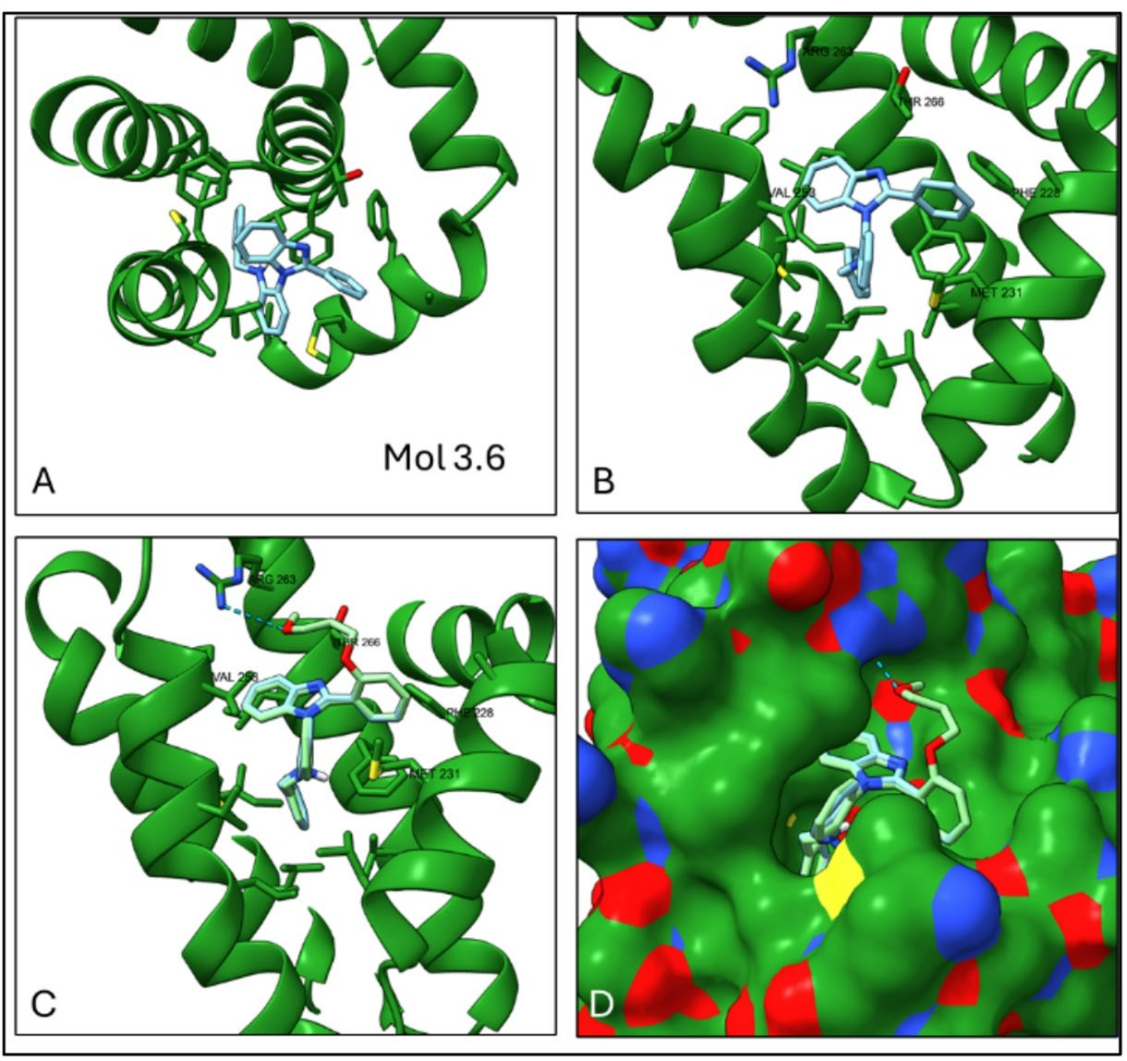
Docking simulation of molecule 3.6 in the MCL1 binding pocket. (**A**) Intercalation of molecule 3.6 with the helices in the MCL1 structure (6FS1). (**B**) The molecule firmly positioned in the hydrophobic pocket, in close proximity to key amino acids MET231 and VAL253. (**C**) Overlap of molecule 3.1 with its linker-modified form (-OCCCOC), highlighting a hydrogen bond to ARG263 (blue dashes). (**D**) Surface representation of MCL1 with both the original (blue) and linker-modified (green) molecules superimposed in the binding pocket.

A QSAR model was built using ~ 1000 compounds with known K_i_ values from ChEMBL. The molecule with the highest estimated docking affinity, Mol 3.6, achieved a modest QSAR prediction of ~ 1800 nM (K_i_). The top scoring conformation for this docked ligand revealed a quinazoline core positioned near the entrance of the binding pocket, with two hydrophobic benzene rings extending into the highly hydrophobic region of the cavity (Fig. 3). Another hydrophobic benzene ring near the entrance appears to interact with surface hydrophobic residues, likely enhancing the overall fit.

An attempt was made to introduce a functional group capable of forming a hydrogen bond with Arg263, a key residue of the MCL1 binding site, while maintaining a good fit within the hydrophobic pocket. Using ChatGPT, 20 linkers were generated to connect the core structure to functional groups that could facilitate this interaction (Appendix A). Three linkers demonstrated significant improvements in QSAR predictions, with predicted K_i_ values below 100 nM. One linker formed a hydrogen bond with Arg263, stabilizing the complex (Fig. 3C). QSAR predicted the K_i_ to improve from ~ 1 µM to 46 nM, while the docking affinity remained favourable at -9.5 kcal/mol (K_d_ ~108 nM), providing a reasonable basis for further exploration.

## Discussion and conclusion

This study evaluated ChatGPT, a general-purpose large language model, for generating novel molecular candidates across three drug discovery tasks. Despite not being trained for drug design, the model proposed chemically coherent molecules with competitive predicted affinities and diverse scaffolds. To address practical feasibility, commercially available analogues were identified and evaluated using QSAR modelling and molecular docking, illustrating how accessible AI can support idea generation and candidate prioritization.

While this study used coding to build machine learning models and perform molecular docking, similar analyses can be conducted using user-friendly platforms such as AutoDock for docking, and ChemSAR or OCHEM for QSAR modelling, which require no coding experience^18,19,33,34^. Even without these tools, researchers can evaluate LLM-generated molecules and analogues using rational design principles.

In task 1, starting from low-affinity EGFR inhibitors, two rounds of QSAR-guided design yielded analogues with predicted IC_50_ values of 20–50 nM, even when their chemical similarity to the generated molecules was modest (as low as 0.6). In the second task, where EGFR was specified, a top analogue achieved an estimated IC₅₀ of 56 nM. In the third task, focusing on MCL1, docking of analogues suggested promising estimated activity (K_d_ ~10–200 nM), but QSAR predictions for these same molecules exceeded 1 µM. Incorporating linkers proposed by ChatGPT restored predicted potency, with one analogue achieving QSAR affinity of 108 nM and a docking score of − 9.5 kcal/mol (K_d_ ~108 nM).

Across all tasks the model reliably produced chemically coherent structures: in task 1 an average of 42.5 SMILES were generated per run with validity averaging ~ 79% across experiments. Tasks 2 and 3 resulted in ~ 87% and ~ 92% valid molecules respectively. Lipinski’s Rule of Five analysis of the featured analogues showed that 2 of 7 task 1 analogues, and 1 of 3 task 2 analogues violated at least one criterion, while all task 3 analogues were fully compliant. Nevertheless, all these candidates were deemed suitable for further exploration as no severe violations or outliers were observed (Table S.7).

Comparison between molecules from tasks 1 and 2 show that both approaches generated promising candidates for EGFR inhibition, though variability between batches and experiments complicates direct ranking. Considering the top three identified analogues, the average predicted activity (IC_50_) in task 2 was 7.10 ± 0.27, ranking second relative to the three task 1 batches (6.55 ± 1.29; 7.61 ± 0.07; 6.76 ± 0.59). Looking beyond averages, two of three task 2 experiments produced analogues with predicted affinities of ~ 7.25 (IC₅₀ ~56 nM), whereas five of nine experiments across all batches in task 1 resulted in molecules with stronger predictions.

Where molecules were generated ab initio, we assessed how much of their architecture reflected known inhibitors versus novel designs. EGFR molecules echoed familiar motifs, particularly nitrogen-rich aromatics, while introducing variation through side-chain designs. Notably, omilancor emerged as an unexpected candidate. MCL1 candidates were generally smaller than well-known inhibitors like Tapotoclax, MI-238, or AZD5991, yet retained recognizable elements, including benzamide and piperazine fragments, alongside more generic ring systems.

A key strength across all tasks was the structural diversity of the identified molecules, spanning multiple scaffolds and functional groups, which broadens the chemical space and supports downstream optimization. Nonetheless, important limitations remain: general-purpose LLMs are not tuned for drug discovery, predicted affinities require experimental validation, and embedded knowledge may lack depth or be outdated.

Future work should integrate LLMs with predictive screening tools, iterative experimental feedback, and specialized databases to improve accuracy and target relevance. Incorporating real-time experimental data could further refine molecular designs and accelerate translation. At the same time, generative AI for molecule design raises ethical and safety concerns, including the risk of “hallucinated” or unsafe structures, inadvertent use of proprietary data, and intellectual property issues. These tools could also be misused to explore toxic or dual-use chemistries, highlighting the need for careful validation, responsible governance, and clear regulatory oversight.

In conclusion, this study demonstrates that accessible, general-purpose LLMs like ChatGPT can support early-stage drug discovery. By rapidly generating and exploring diverse molecular hypotheses, and identifying feasible analogues, this approach offers a practical pathway to democratize and accelerate candidate discovery.

## Supplementary Information

Below is the link to the electronic supplementary material.

Supplementary Material 1

Supplementary Material 2

Supplementary Material 3

## Data Availability

ChEMBL datasets can be downloaded from https://www.ebi.ac.uk/chembl/, structures for EGFR (3BEL) and MCL1 (6FS1) are available at https://www.rcsb.org/.

## Code availability

Code and processed data are available in the github repository: https://github.com/abbiAR/AI-in-Early-Stage-Drug-Discovery-and-Ideation.
