# Supplementary material for: A case study of the application of AI to early stage drug discovery
Source: Sci Rep. 2025 Dec 26;16:2902. doi: 10.1038/s41598-025-32805-1 (PMC12827298; doi:10.1038/s41598-025-32805-1)
Supplement: Supplementary file 3 — Supplementary Material 3 [file 41598_2025_32805_MOESM3_ESM.pdf]

Exploring the Utility of General-Purpose AI in Early-Stage Drug Discovery and Ideation: A Case Study with ChatGPT

Supplementary Materials

Batch 1	Batch 2	Batch 3
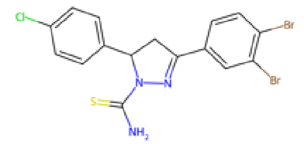 b.1-1	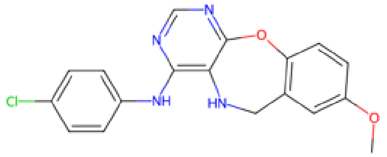 b.2-1	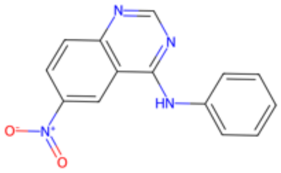 b.3-1
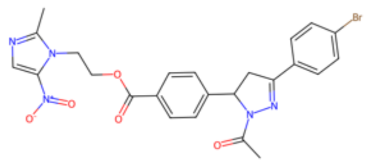 b.1-2	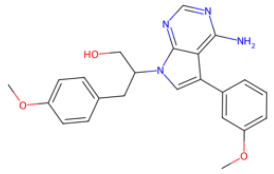 b.2-2	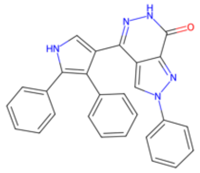 b.3-2
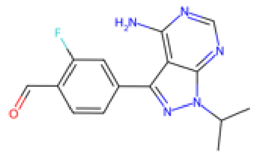 b.1-3	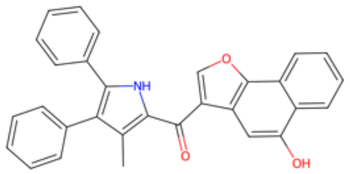 b.2-3	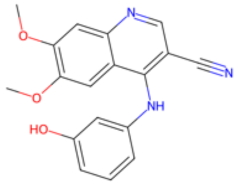 b.3-3
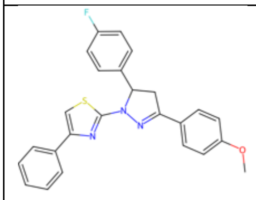 b.1-4	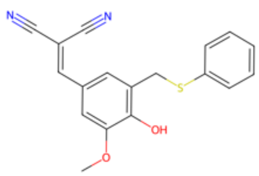 b.2-4	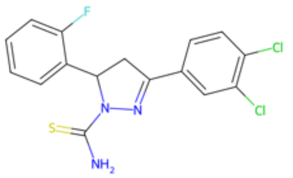 b.3-4
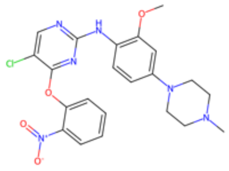 b.1-5	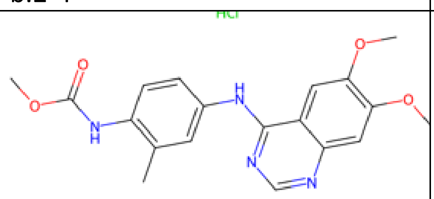 b.2-5	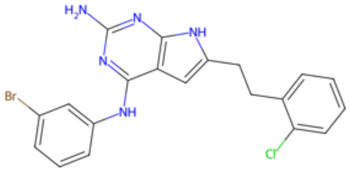 b.3-5

Table S1. Randomly selected batches of low affinity molecules for task 1. Five molecules of low affinity (IC50 = 10 – 3.16 µM) were selected randomly from the dataset and provided to GPT4o for optimisation in task 1.

Molecule	SMILES	pChembl	IC50 (µM)
b.1-1	C1C(N(N=C1C2=CC(=C(C=C2)Br)Br)C(=S)N)C3=CC=C(C=C3)Cl	5.09	8.13
b.1-2	CC1=NC=C(N1CCOC(=O)C2=CC=C(C=C2)C3CC(=NN3C(=O)C)C4=CC=C(C=C4)Br)[N+](=O)[O-]	5.38	4.17
b.1-3	CC(C)N1C2=NC=NC(=C2C(=N1)C3=CC(=C(C=C3)C=O)F)N	5.05	8.91

b.1.4	COC1=CC=C(C=C1)C2=NN(C(C2)C3=CC=C(C=C3)F)C4=NC(=CS4)C5=CC=CC=C5	5.24	5.75
b.1-5	CN1CCN(CC1)C2=CC(=C(C=C2)NC3=NC=C(C(=N3)OC4=CC=CC=C4[N+](=O)[O-])Cl)OC	5.12	7.59
b.2-1	COC1=CC2=C(C=C1)OC3=NC=NC(=C3NC2)NC4=CC=C(C=C4)Cl	5.24	5.75
b.2-2	COC1=CC=C(C=C1)CC(CO)N2C=C(C3=C(N=CN=C32)N)C4=CC(=CC=C4)OC	5.01	9.77
b.2-3	CC1=C(NC(=C1C2=CC=CC=C2)C3=CC=CC=C3)C(=O)C4=COC5=C4C=C(C6=CC=CC=C65)O	5.29	5.13
b.2.4	COC1=CC(=CC(=C1O)CSC2=CC=CC=C2)C=C(C#N)C#N	5.22	6.03
b.2-5	CC1=C(C=CC(=C1)NC2=NC=NC3=CC(=C(C=C32)OC)OC)NC(=O)OC.Cl	5.4	3.98
b.3-1	C1=CC=C(C=C1)NC2=NC=NC3=C2C=C(C=C3)[N+](=O)[O-]	5.3	5.01
b.3-2	C1=CC=C(C=C1)C2=C(NC=C2C3=NNC(=O)C4=NN(C=C34)C5=CC=CC=C5)C6=CC=CC=C6	5.44	3.63
b.3-3	COC1=C(C=C2C(=C1)C(=C(C=N2)C#N)NC3=CC(=CC=C3)O)OC	5.2	6.31
b.3.4	C1C(N(N=C1C2=CC(=C(C=C2)Cl)Cl)C(=S)N)C3=CC=CC=C3F	5.18	6.61
b.3-5	C1=CC=C(C(=C1)CCC2=CC3=C(N2)N=C(N=C3NC4=CC(=CC=C4)Br)N)Cl	5.47	3.39

Table S2. SMILES strings and activities for molecules in initial batches. Each molecule is named according to its batch and a number between 1-5 (for instance batch1, molecule 1 = b.1-1). The measured activity is also declared, both as pChembl value which was used to train the machine learning models, as well as the corresponding IC50 value.

Experiment	TOP Generated (BATCH 1)	TOP Generated (BATCH 2)	TOP Generated (BATCH 3)
Exp. 1	MOL 1.1<br>32 nM 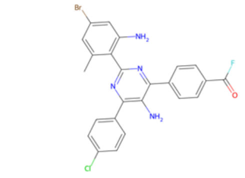	MOL 1.2<br>52 nM 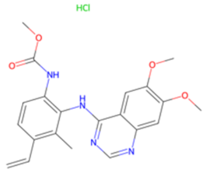	MOL 1.3<br>105 nM 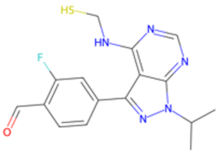
Exp. 2	MOL 1.4<br>330 nM 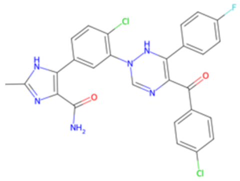	MOL 1.5<br>48 nM 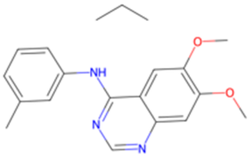	MOL 1.6<br>113 nM 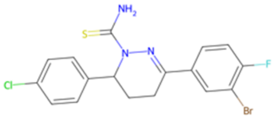
Exp. 3	MOL 1.7<br>52 nM 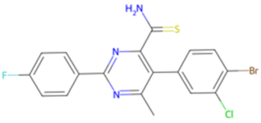	MOL 1.8<br>24 nM 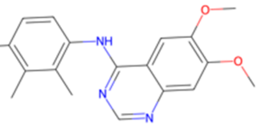	MOL 1.9<br>11 nM 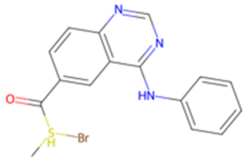

Table S3. ChatGPT (GPT-4o) generated molecules with predicted top activity per initial batch (1-3) and experiment (1-3) at after two iterations.

Molecule	SMILES	pChembl	IC50 (nM)
MOL 1.1	CC1=CC(=CC(=C1C2=NC(=C(C(=N2)C3=CC=C(C=C3)C(=O)F)N)C4=CC=C(C=C4)Cl)N)Br	7.492	32
MOL 1.2	CC1=C(C=CC(=C1NC2=NC=NC3=CC(=C(C=C32)OC)OC)NC(=O)OC.Cl)C=C	7.287	52
MOL 1.3	CC(C)N1C2=NC=NC(=C2C(=N1)C3=CC(=C(C=C3)C=O)F)NCS	6.979	105
MOL 1.4	CC1=NC(=C(N1)C2=CC(=C(C=C2)Cl)N3C=NC(=C(N3)C4=CC=C(C=C4)F)C(=O)C5=CC=C(C=C5)Cl)C(=O)N	6.481	330
MOL 1.5	CC1=CC=CC(=C1)NC2=NC=NC3=CC(=C(C=C32)OC)OC.C(C)(C)	7.315	48
MOL 1.6	C1CC(N(N=C1C2=CC(=C(C=C2)F)Br)C(=S)N)C3=CC=C(C=C3)Cl	6.947	113
MOL 1.7	CC1=NC(=NC(=C1C2=CC(=C(C=C2)Br)Cl)C(=S)N)C3=CC=C(C=C3)F	7.281	52
MOL 1.8	CC1=C(C=CC(=C1C)NC2=NC=NC3=CC(=C(C=C32)OC)OC)C	7.624	24
MOL 1.9	C1=CC=C(C=C1)NC2=NC=NC3=C2C=C(C=C3)C(=O)S(C)Br	7.950	11
MOL 1.10	Cl.COCCOc1cc2ncnc(Nc3cccc(C=C)c3)c2cc1OCCOC	7.517	30
MOL 1.11	CC(=O)c1ccc(Nc2ncnc3ccccc23)cc1	7.279	52
MOL 1.12	COc1cc2ncnc(Nc3ccc(Cl)cc3C)c2cc1OC	7.665	22
MOL 1.13	CC1=NC(=NC(=C1C2=CC(=C(C=C2)F)O)C(=S)N)C3=CC=C(C=C3)Br	7.665	87
MOL 1.14	C1CC(N(N=C1C2=CC(=C(C=C2)F)Br)C(=S)N)C3=CC=C(C=C3)Cl	6.947	113
MOL 1.15	C1=CC(=C(C=C1)C(=O)N)NC2=NC=NC(=C2)C3=CC=CC=C3	7.266	54
MOL 1.16	COc1cc(CN2CCC(CC2)c2nc(ncc2-c2cccc(C)c2)-c2ccncc2)ccc1F	6.449	356
MOL 1.17	CC(=O)Nc1ccc(cc1)-c1cc(nc(n1)-c1ccc(Br)cc1)-c1ccc(NC(C)=O)cc1	7.397	40
MOL 1.18	NC(=S)N1N=C(CC1c1ccc(Cl)cc1)c1cccnc1	6.708	196
MOL 1.19	Cl.Nc1ccc(Nc2cc(ncn2)-c2ccccc2)cc1	7.327	47

Table S4. SMILES and activities for generated molecules by ChatGPT that features in task 1. The QSAR predicted activity is shown as a pChembl value which the machine learning models were trained on, along with the corresponding IC50 value.

Molecule	SMILES	pChembl	IC50 (nM)
MOL 2.1	C1=CN=C2C(=N1)N=CC(=N2)C3=CC=C(C=C3)C(=O)N4CCCC4	7.025	94
MOL 2.2	CC1=CN=C(N=C1)C2=NC(=CC=C2)C(=O)NCCS(=O)(=O)N	6.937	116
MOL 2.3	C1=CC2=CC=CC=C2N=C1C3=CC(OCC4)C4CN3	6.471	338
MOL 2.4	CC(N(C)C)c1ccc(cc1)-c1cnc2nccnc2n1	7.257	55
MOL 2.5	O=C(N1CCN(CC1)C(=O)c1cccc(n1)-c1nc2ccccc2[nH]1)c1cccc(n1)-c1nc2ccccc2[nH]1	7.254	56
MOL 2.6	O=C(NC1CC1)[C@@]12CCO[C@@H]1CCN(Cc1ccc3ccccc3n1)C2	6.783	165

Table S5. SMILES and activities for generated molecules by ChatGPT that features in task 2. The QSAR predicted activity is shown as a pChembl value which the machine learning models were trained on, along with the corresponding IC50 value.

Molecule	SMILES	Kd (nM)
MOL 3.1	C1=CC2=C(C=C1F)C3=CC=CC=C3N2CCOC4=CC=CC=C4	39
MOL 3.2	C1=C(C=C(C=C1)C2=CN=CC=C2)C3=CN(C4=CC=CC=C4)C=C3	108
MOL 3.3	CN1CCN(CC1)C2=CC=C(C=C2)C(=O)NC3=CC=CC=C3N4C=NC5=CC=CC=C54	91

MOL 3.4	O=C(COc1ccccc1)n1c2ccccc2c2ccccc12	20
MOL 3.5	[Cl-].c1ccc(cc1)-c1ccc[n+](c1)-c1ccccc1	210
MOL 3.6	C(Nc1ccccc1-n1c(nc2ccccc12)-c1ccccc1)c1ccccc1	12

Table S6. SMILES and activities for top generated molecules by ChatGPT in task 3. The estimated affinity from Autodock VINA is shown (Kd).

molecule	smiles	mw	logp	H-don	H-acc
MOL 1.1	CC1=CC(=CC(=C1C2=NC(=C(C(=N2)C3=CC=C(C=C3)C(=O)F)N)C4=CC=C(C=C4)Cl)N)Br	511.78	6.48	2	5
MOL 1.2	CC1=C(C=CC(=C1NC2=NC=NC3=CC(=C(C=C32)OC)OC)NC(=O)OC.Cl)C=C	430.89	4.94	2	7
MOL 1.3	CC(C)N1C2=NC=NC(=C2C(=N1)C3=CC(=C(C=C3)C=O)F)NCS	345.4	3.33	2	7
MOL 1.4	CC1=NC(=C(N1)C2=CC(=C(C=C2)Cl)N3C=NC(=C(N3)C4=CC=C(C=C4)F)C(=O)C5=CC=C(C=C5)Cl)C(=O)N	549.39	5.53	3	6
MOL 1.5	CC1=CC=CC(=C1)NC2=NC=NC3=CC(=C(C=C32)OC)OC.C(C)(C)	339.44	5.12	1	5
MOL 1.6	C1CC(N(N=C1C2=CC(=C(C=C2)F)Br)C(=S)N)C3=CC=C(C=C3)Cl	426.74	5.03	1	2
MOL 1.7	CC1=NC(=NC(=C1C2=CC(=C(C=C2)Br)Cl)C(=S)N)C3=CC=C(C=C3)F	436.74	5.31	1	3
MOL 1.8	CC1=C(C=CC(=C1C)NC2=NC=NC3=CC(=C(C=C32)OC)OC)C	323.4	4.32	1	5
MOL 1.9	C1=CC=C(C=C1)NC2=NC=NC3=C2C=C(C=C3)C(=O)S(C)Br	376.28	4.45	2	4
**MOL 1.10**	**Cl.COCCOc1cc2ncnc(Nc3cccc(C=C)c3)c2cc1OCCOC**	**431.92**	**4.49**	**1**	**7**
**MOL 1.11**	**CC(=O)c1ccc(Nc2ncnc3ccccc23)cc1**	**263.3**	**3.58**	**1**	**4**
**MOL 1.12**	**COc1cc2ncnc(Nc3ccc(Cl)cc3C)c2cc1OC**	**329.79**	**4.35**	**1**	**5**
MOL 1.13	CC1=NC(=NC(=C1C2=CC(=C(C=C2)F)O)C(=S)N)C3=CC=C(C=C3)Br	418.29	4.36	2	4
MOL 1.14	C1CC(N(N=C1C2=CC(=C(C=C2)F)Br)C(=S)N)C3=CC=C(C=C3)Cl	426.74	5.03	1	2
MOL 1.15	C1=CC(=C(C=C1)C(=O)N)NC2=NC=NC(=C2)C3=CC=CC=C3	290.33	2.99	2	4
**MOL 1.16**	**COc1cc(CN2CCC(CC2)c2nc(ncc2-c2cccc(C)c2)-c2ccncc2)ccc1F**	**468.58**	**6.04**	**0**	**5**
**MOL 1.17**	**CC(=O)Nc1ccc(cc1)-c1cc(nc(n1)-c1ccc(Br)cc1)-c1ccc(NC(C)=O)cc1**	**501.38**	**6.16**	**2**	**4**
**MOL 1.18**	**NC(=S)N1N=C(CC1c1ccc(Cl)cc1)c1cccnc1**	**316.82**	**3.13**	**1**	**3**
**MOL 1.19**	**Cl.Nc1ccc(Nc2cc(ncn2)-c2ccccc2)cc1**	**298.78**	**3.89**	**2**	**4**
MOL 2.1	C1=CN=C2C(=N1)N=CC(=N2)C3=CC=C(C=C3)C(=O)N4CCCC4	305.34	2.32	0	5
MOL 2.2	CC1=CN=C(N=C1)C2=NC(=CC=C2)C(=O)NCCS(=O)(=O)N	321.36	-0.13	2	6
MOL 2.3	C1=CC2=CC=CC=C2N=C1C3=CC(OCC4)C4CN3	252.32	2.58	1	3

**MOL 2.4**	**CC(N(C)C)c1ccc(cc1)-c1cnc2nccnc2n1**	**279.35**	**2.71**	**0**	**5**
**MOL 2.5**	**O=C(N1CCN(CC1)C(=O)c1cccc(n1)-c1nc2ccccc2[nH]1)c1cccc(n1)-c1nc2ccccc2[nH]1**	**528.58**	**4.16**	**2**	**6**
**MOL 2.6**	**O=C(NC1CC1)[C@@]12CCO[C@@H]1CCN(Cc1ccc3ccccc3n1)C2**	**351.45**	**2.49**	**1**	**4**
MOL 3.1	C1=CC2=C(C=C1F)C3=CC=CC=C3N2CCOC4=CC=CC=C4	305.35	5.01	0	2
MOL 3.2	C1=C(C=C(C=C1)C2=CN=CC=C2)C3=CN(C4=CC=CC=C4)C=C3	296.37	5.21	0	2
MOL 3.3	CN1CCN(CC1)C2=CC=C(C=C2)C(=O)NC3=CC=CC=C3N4C=NC5=CC=CC=C54	411.51	4.03	1	5
**MOL 3.4**	**O=C(COc1ccccc1)n1c2ccccc2c2ccccc12**	**301.35**	**4.51**	**0**	**3**
**MOL 3.5**	**[Cl-].c1ccc(cc1)-c1ccc[n+](c1)-c1ccccc1**	**267.76**	**0.63**	**0**	**0**
**MOL 3.6**	**C(Nc1ccccc1-n1c(nc2ccccc12)-c1ccccc1)c1ccccc1**	**375.48**	**6.3**	**1**	**3**

Table S7. Lipinski's rule of 5 for the molecules featuring in the manuscript. Molecular weight, logP, H-donors and H-acceptors were calculated using RDKit (Descriptors.MolWt(mol), Crippen.MolLogP(mol), Lipinski.NumHDonors(mol), Lipinski.NumHAcceptors(mol)). Values in red indicate rule-breaks. Featured analogues are highlighted in bold.
